# Long-term maintenance treatment of recurrent ureteropelvic junction obstruction with covered metallic ureteral stent

**DOI:** 10.1097/MD.0000000000033363

**Published:** 2023-03-31

**Authors:** Xinwei Tang, Mingrui Wang, Haopu Hu, Chin-Hui Lai, Qi Wang, Kexin Xu, Tao Xu, Hao Hu

**Affiliations:** a Department of Urology, Peking University People’s Hospital, Beijing, China.

**Keywords:** covered metallic ureteral stent, maintenance treatment, pyeloplasty, recurrent ureteropelvic junction obstruction

## Abstract

Whether or not the covered metallic ureteral stent can be used as maintenance treatment for recurrent ureteropelvic junction obstruction (UPJO) after pyeloplasty is unknown. Therefore, this study aims to analyze its feasibility. We retrospectively analyzed the records of 20 patients with recurrent UPJO who were treated with the covered metallic ureteral stents from March 2019 to June 2021 at our institution. Then, we assessed their renal function, stent patency and stent-related quality of life by the blood creatinine, renal ultrasound (or computed tomography), and the Chinese version of the ureteral symptom score questionnaire (USSQ). The last follow-up mean blood creatinine dropped from 0.98 ± 0.22 to 0.91 ± 0.21 mg/dL (*P =* .04), and the median renal pelvic width was reduced from 3.25 (3.10) to 2.00 (1.67) cm (*P* = .03) compared with the preoperative conditions. Meanwhile, the last follow-up mean USSQ total score of the covered metallic ureteral stent among the 16 patients with preoperative indwelling double-J ureteral stent was 78.56 ± 14.75, significantly lower than the preoperative USSQ total score, which was 102.25 ± 5.57 (*P* < .001). During the median duration of follow-up of 27.00 (18.00) months, 85% (17/20) of patients maintained unobstructed drainage from the renal pelvis to the ureter. Stent-related complications occurred in 7 patients, 3 of which failed because of complications, including stent migration (1 patient), stent encrustation (1 patient), and stent-related infection (1 patient). The covered metallic ureteral stent is feasible for the long-term maintenance treatment of recurrent UPJO after pyeloplasty.

## 1. Introduction

Recurrent ureteropelvic junction obstruction (UPJO) after pyeloplasty is a rare but severe complication that often requires a second operation. Approximately 11.4% of recurrent UPJO required reoperations.^[1]^ Although several studies have shown that the second pyeloplasty surgeries, including the open, laparoscopic, and robot-assisted laparoscopic approaches, all displayed a high success rate (>90%) in the treatment of recurrent UPJO.^[2]^ However, there is still no consensus on the best surgical approach for recurrent UPJO. In addition, second-time pyeloplasty is more difficult than first-time pyeloplasty,^[3]^ of which can only be carried out in a few advanced medical centers. On the one hand, for patients with recurrent UPJO who are unable or reluctant to receive a second pyeloplasty because of physical condition and/or loss of confidence after the first-time pyeloplasty, the second-time pyeloplasty can no longer satisfy the clinical needs of those patients. On the other hand, maintenance therapy that maintains smooth drainage of urine through the long-term indwelling ureteral stent or nephrostomy is also common in clinical practice and may be more in line with the treatment expectations of the above-mentioned patients.

The polymer double-J ureteral stent is the most commonly used for the maintenance treatment of recurrent UPJO in clinical practice. However, it may come with various problems such as moderate dilation and drainage, high complication rate, and frequent stent replacement in a short period. Therefore, the placement of a double-J ureteral stent sometimes fails to improve the patient’s urine drainage, but instead, undermines the patient’s quality of life.^[4]^ The placement of the covered metallic ureteral stent results in a good drainage outcome and better quality of life in the maintenance treatment of benign, iatrogenic, and malignant ureteral strictures.^[5–7]^ However, there is no relevant research on the outcomes of the covered metallic ureteral stent placement in the long-term maintenance treatment of recurrent UPJO. Therefore, in this study, we analyzed the feasibility of the new segmental-covered metallic ureteral stent for the long-term maintenance treatment of recurrent UPJO after pyeloplasty.

## 2. Materials and methods

Study design and patients: This study included 20 patients with recurrent UPJO who were treated with segmental covered metallic ureteral stents at our institution from March 2019 to June 2021 (Table 1), of which 1 patient had experienced 2 failures of pyeloplasty and 2 failures of endoscopic treatment, 2 patients had experienced 2 failures of pyeloplasty, 8 patients had experienced 1 failure of pyeloplasty and at least 1 failure of endoscopic treatment, and 9 patients had experienced 1 failure of pyeloplasty. All these patients were diagnosed as recurrent UPJO by intraoperative anterograde/retrograde urography. The success of the covered metallic ureteral stent in the long-term maintenance treatment of recurrent UPJO is the smooth drainage of urine from the renal pelvis to the ureter during the follow-up; the failure of the stent treatment was the increase of hydronephrosis after stent placement, the suspected deterioration of post-renal renal function and the need to change to other surgical procedures (such as double-J stent placement, nephrostomy, or pyeloplasty). The stent position, renal function, and stent patency were evaluated by abdominal X-ray, blood creatinine, and renal ultrasound (or computed tomography). The stent-related quality of life of patients was assessed by the Chinese version of the ureteral symptom score questionnaire (USSQ) (see Questionnaire 1, Supplemental Digital Content, http://links.lww.com/MD/I704, the Chinese version of the USSQ). The original English version of the USSQ was a validated, widely applied questionnaire for stent-related symptoms (see Questionnaire 2, Supplemental Digital Content, http://links.lww.com/MD/I705, the original English version of the USSQ). It analyzes various domains of health affected by ureteral stents, which consists of 38 items separated into 6 following sections: urinary symptoms, pain, general health, work performance, sexual matters, and additional problems. The scoring system for the questionnaire consists of a simple sum of the scores for individual questions in each section. Each section had a summary (index) score except for the questions on additional problems. The high scores indicated worse outcomes.^[8]^ Up to date, the original English version of the USSQ has been translated into different languages, and validated translations are available in Italian,^[9]^ French,^[10]^ Korean,^[11]^ Spanish,^[12]^Arabic,^[13]^ German,^[14]^ Chinese,^[15]^ and Danish.^[16]^ The current results demonstrate that the Chinese version of the USSQ is a reliable and valid instrument for measuring the symptom complex in male and female patients with an indwelling ureteral stent.^[15]^ The study was approved by the Ethics Review Committee of Peking University People’s Hospital and individual consent for this retrospective analysis was waived on April 7, 2022 (no: 2022PHB980).

**Table 1:** Basic clinical characteristics of the covered metallic ureteral stent.

Characteristics	
Age, yr	27.50 (12.00)
Gender, n	
Male	15.00
Female	5.00
Side, n	
Right	8.00
Left	12.00
Causes of strictures, n	
Recurrent UPJO	20.00
Stricture length (cm)	
≤2	15.00
>2	5.00
Previous D-J indwelling, n	
Single D-J	12.00
Second D-J	4.00
Indwelling time, mo	13.50 (39.00)
Replacement frequency, mo/time	5.00 (3.00)

D-J = double-J stent, UPJO = ureteropelvic junction obstruction.

**Figure 1.**
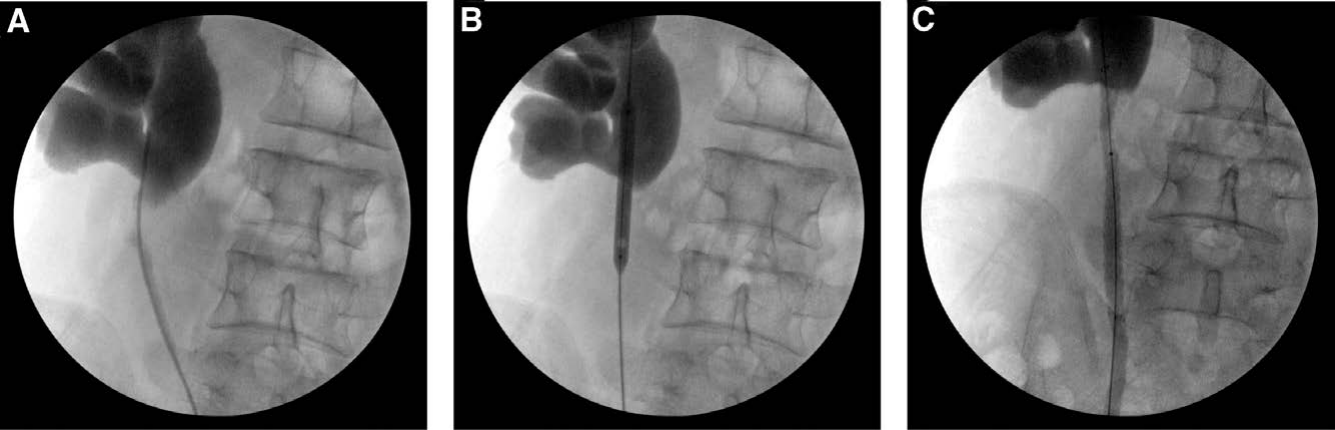
Steps of covered metallic ureteral stent insertion for the treatment of the recurrent ureteropelvic junction obstruction. A. Location and length of the ureteropelvic junction stricture were confirmed by retrograde urography; B. Ureteropelvic junction stricture was dilated by a balloon; C. Covered metallic ureteral stent was released in the correct placement.

Surgical Method: Retrograde urography was performed to confirm the location and length of the stenosis (Fig. 1A). Safety and operation guidewires were inserted through the ureteral catheter. A balloon dilatation catheter (21 F, 6 cm, BARD, New Jersey, USA) was pushed along the operation guidewire to be placed in the narrow segment under X-ray fluoroscopy, and then the balloon dilatation was inflated with a pressure of 25ATM for 3 minutes. The narrow segment had been dilated well as seen through X-ray fluoroscopy (Fig. 1B). After the balloon catheter was withdrawn, the covered metallic ureteral stent delivery catheter (21 F, 12 cm, Allium Medical Solutions Ltd., Caesarea, Israel) was pushed along the operation guidewire. Through X-ray fluoroscopy, the upper head of the stent was located in the renal pelvis, and the head was approximately 0.5 to 1.0 cm away from the proximal end of the stenosis. The tail of the stent was located below the stenosis and at the place where there was no stenosis. Then, the stent was slowly released after the end of the delivery device was fixed, and the stent did not migrate under X-ray fluoroscopy. After full expansion for approximately 3 minutes, the delivery device was withdrawn (Fig. 1C). The ureteral catheter was again pushed along the guidewire, and retrograde urography was performed to confirm that the ureter was unobstructed and the stent position was ideal. Finally, the catheter and guidewires were withdrawn, and the operation was done.

### 2.1. Statistical analysis

IBM SPSS Statistics for Windows, Version 25.0 (IBM Corp., Armonk, NY) was used for data processing. Normally distributed measurement data were represented by mean ± standard deviation, and measurement data that did not conform to normal distribution were represented by median (interquartile range). Paired-sample *t* test or rank-sum test was used to compare the differences in measurement data. *P* < .05 was considered statistically significant.

## 3. Results

In this study, all 20 patients (15 males and 5 females, median age of 27.50 (12.00) years) had recurrent UPJO after pyeloplasty which resulted in unilateral recurrent stenosis. A total of 12 patients displayed stenosis on the left side and the other 8 suffered from recurrent UPJO on the right side (Table 1). The preoperative average blood creatinine level and median renal pelvis width were 0.98 ± 0.22 mg/dL and 3.25 (3.10) cm, respectively. Of the 20 patients, 16 had already indwelled with long-term double-J ureteral stents before the placement of the covered metallic ureteral stent. The median double-J stents indwelling time and replacement time were 13.50 (39.00) and 5.00 (3.00) months, respectively. The preoperative mean USSQ total score of those 16 patients was 102.25 ± 5.57 points. Particularly, the preoperative median urinary system symptoms score (U) was 33.50 (2.00) points; the preoperative median body pain score (P) was 19.50 (3.00) points; the preoperative mean general health score (G) was 19.38 ± 2.42 points; the preoperative median work performance score (W) was 12.00 (1.00) points; the preoperative median sexual matters score (S) was 6.00 (5.00) points (Table 2).

**Table 2:** Ureteral stent symptoms evaluation of the previous double-J stenting patients.

Score	Covered metallic ureteral stent		*P* value
	Preoperative	Postoperative	
USSQ total score	102.25 ± 5.57	78.56 ± 14.75	<.001
USSQ urinary symptoms score	33.50 (2.00)	24.63 ± 5.10	.001
USSQ body pain score	19.50 (3.00)	11.50 (11.00)	<.001
USSQ general health score	19.38 ± 2.42	14.44 ± 2.76	<.001
USSQ work performance score	12.00 (1.00)	11.31 ± 2.18	.34
USSQ sexual matters score	6.00 (5.00)	3.50 (5.00)	.42

USSQ = ureteral stent symptoms questionnaire.

The covered metallic ureteral stent placement surgeries were successfully conducted in all 20 patients with a median operation time of 45.00 (23.00) minutes. The mean blood creatinine level on the first postoperative day was 0.91 ± 0.19 mg/dL, lower than the mean preoperative blood creatinine (*P* = .03) value. The median postoperative hospital stay was 3.00 (4.00) days. During the postoperative hospitalization, 4 patients experienced severe pain, and 2 patients developed infection-caused fever, but they all resolved after symptomatic treatment (Table 3).

**Table 3:** Numbers of all types of complications.

Complications	Total
Perioperative complications	
Severe pain	4
Fever	2
Long-term stent-related complications	
Stent migration	5
Stent encrustation	1
Stent related infection	1

The postoperative median follow-up time was 27.00 (18.00) months, and 85% (17/20) of the patients maintained a smooth flow of urine from the renal pelvis to the ureter during the follow-up (Table 4). The mean blood creatinine level at the last follow-up was 0.91 ± 0.21 mg/dL, significantly lower than that before the operation (0.98 ± 0.22 mg/dL, *P* = .04). The median renal pelvis width at the last follow-up 2.00 (1.67) cm was significantly reduced (*P =* .03) as compared with the preoperative median renal pelvis width 3.25 (3.10) cm. Among those 16 patients with preoperative indwelling polymer double-J ureteral stent, the mean USSQ total score at the last follow-up significantly reduced to 78.56 ± 14.75 (*P* < .001). Particularly, the mean urinary system symptoms score (U) at the last follow-up was 24.63 ± 5.10 points, the median body pain score (P) at the last follow-up was 11.50 (11.00) points, the mean general health score (G) at the last follow-up was 14.44 ± 2.76 points, and all of the above were significantly lower than those before the operation. In addition, the mean work performance score (W) and the median sexual matters score (S) at the last follow-up were not significantly different from those before the operation (Table 2).

**Table 4:** Long-term treatment outcomes of the covered metallic ureteral stent.

	Covered metallic ureteral stent
Follow-up months	27.00 (18.00)
Success rate*, percent	85%
Cause of failure, n	
Stent migration	1
Stent encrustation	1
Stent related infection	1

* Success rate is an estimate, and the standard error is omitted.

Stent migration (5 patients) was the most common long-term stent-related complication during follow-up, 4 of which continued to indwell the covered metallic ureteral stents after adjusting, and 1 patient had the stent removed because of repeated stent migration, and then underwent a second ureteroplasty because of increased hydronephrosis. The mean ureteral stent migration time was 5.60 ± 6.50 months. Other stent-related complications included stent encrustation (1 patient) and stent infection (1 patient). In the stent encrustation patient, we changed the covered metallic ureteral stent to nephrostomy after the stent was pulled out. In another stent related infection patient, we replaced the covered metallic ureteral stent with a double-J ureteral stent (Tables 3 and 4).

## 4. Discussion

Although the success rate of pyeloplasty in the treatment of UPJO is very high (>94%), the failure of pyeloplasty may require a second surgical intervention.^[1]^ The main intervention indications include worsening asymptomatic hydronephrosis (59%), pain (26%), urosepsis (7.5%), and others (7.5%).^[17]^ The most common treatments are ureteral stent maintenance, followed by pyeloplasty or endoscopic treatment, and few patients require nephrectomy or kidney transplantation.^[1]^ The maintenance treatment by placing a ureteral stent has the advantages of simplicity, fast operation, low bleeding risk, and relatively low cost. Even if the first ureteral stent drainage treatment fails, the ureteral stent can also be replaced or switched to other treatments, such as pyeloplasty and endoscopy treatment. Therefore, the ureteral stent placement treatment is more acceptable to both the patient and the urologist.

The current clinical maintenance treatment for recurrent UPJO primarily includes polymer double-J ureteral stent placement and nephrostomy. The polymer double-J ureteral stent is the most used one for maintenance treatment of recurrent UPJO. However, the polymer double-J stent sometimes fails to provide sufficient dilation and urine drainage for the stenosis of the ureter because of the mediocre mechanical properties and thinner lumen. It also inhibits ureteral peristalsis, restricts urine drainage, and causes complications such as stent related infection, stent encrustation, and urinary tract irritation. Moreover, the double-J stent requires frequent replacement assisted by the cystoscope (every 3–6 months).^[4]^ These problems severely affect patients’ quality of life and limit the efficacy of ureteral stent maintenance treatment for recurrent UPJO. Similarly, nephrostomy severely affects patients’ quality of life and may cause complex complications, such as infection, bleeding, skin damage, catheter blockage, and accidental displacement, which require frequent nephrostomy replacement and bring inconveniences and additional costs to patients.^[18]^

In recent years, a novel self-expanding ureteral stent came into a stage that is metallically covered, segmentally designed, with large-caliber lumen, and long-term indwelling time. This one provides a powerful dilation, thereby leading to smoother urine drainage and higher quality of life as compared with the traditional polymer double-J ureteral stent.^[19]^ Up to date, the largest prospective study of the covered metallic ureteral stent for ureteral stricture indicated that the covered metallic ureteral stent is effective in relieving ureteral obstruction. The overall surgical success rate was 73.2% over a follow-up of 15 months.^[20]^ Furthermore, covered metallic ureteral stent was originally used in treating malignant ureteral strictures, however, it also works well in benign and iatrogenic ureteral strictures, which may be caused by retroperitoneal fibrosis, ureteroscopic lithotripsy, and ureteroileal anastomotic stenosis.^[5–7,21,22]^ Nevertheless, whether or not it can be used for the long-term maintenance treatment of recurrent UPJO has not been investigated.

Therefore, the long-term effects of the covered metallic ureteral stent placement were evaluated instead of the traditional double-J ureteral stent or nephrostomy for maintenance treatment of recurrent UPJO. In our study: 80% (16/20) of patients with recurrent UPJO had already been placed by long-term indwelling double-J ureteral stents. The median double-J stents indwelling time and replacement time were 13.50 (39.00) and 5.00 (3.00) months, respectively. There are primary two reasons why these patients with recurrent UPJO choose ureteral stent maintenance treatment: first, they have lost confidence in pyeloplasty and are reluctant to undergo secondary pyeloplasty, particularly those who have experienced multiple failures of pyeloplasty; second, multiple pyeloplasties and repeated endoscopic surgery have not solved the patient’s UPJO, and the patient can only maintain urine drainage from the kidney through the long-term placement of ureteral stent or nephrostomy until a new treatment method appears.

However, for these patients with recurrent UPJO who are unwilling or intolerant to secondary pyeloplasty and suffer from frequent replacements of double-J ureteral stents for a long time, the success rate of long-term maintenance treatment of recurrent UPJO with covered metallic ureteral stents is 85% (17/20) with a median follow-up of 27.00 (18.00) months. During the follow-up, we found that among the 16 patients who had indwelled double-J ureteral stent before the surgery, the USSQ total score, urinary symptoms score (U), body pain score (P), and general health score (G) at the last follow-up were all significantly lower than that before the surgery (Table 2). This suggests that long-term indwelling-covered metallic ureteral stents can help reduce stent-related discomfort and improve patients’ quality of life as compared with double-J ureteral stents. These benefits may have resulted from the following reasons: first, recurrent UPJO always comes with segmental stenosis, and the large-caliber segmental design of the covered metallic ureteral stent can not only ensure adequate renal urine drainage but also minimize the irritation of the normal segment of ureter wall; second, the segmental covered metallic stent has no double-J structure, hence, that there is only 0.5 to 1 cm soft and elastic tubular end exposed to the internal renal pelvis after the surgery. The new one reduces the irritation to the renal pelvis and bladder urothelial tissue as compared with the traditional double-J stent; third, the indwelling time of the coated metallic ureteral stent is much longer,^[23]^ which significantly reduces the frequencies of stent replacement and patients’ hospitalization, thereby relieving the psychological, physical, and economic burden from patients.

The postoperative stent-related complications of covered metallic ureteral stents remain the main factors affecting the outcomes of maintenance treatment of recurrent UPJO (Table 4). Stent migration was the most common postoperative long-term stent-related complication in this study, accounting for 5 (25%) stent-related complications. However, only 1 patient eventually had the stent removed because of repeated stent migration and then underwent a second ureteroplasty because of increased hydronephrosis. The other 4 cases continued to indwell the covered metallic ureteral stent after adjusting the stent (Tables 3 and 4). A previous multicenter study has reported that the covered metallic ureteral stent migration rate was associated with the etiology of ureteral stricture. During a mean follow-up of 17 months, the overall stent migration rate is 14.29% (7/49), and the stent migration rates with different etiologies were stricture following the surgery/radiation therapy for gynecologic malignancy 12.00% (3/25), stricture following surgical and topical treatment for bladder cancer 12.50% (1/8), ureteroenteric anastomosis stricture after urinary diversion 20.00% (1/5), stricture following endoscopic treatment of ureteral calculi 0% (0/6), ureterocutaneostomy stricture 100% (2/2), and stricture following renal transplant 0% (0/3).^[24]^ Meanwhile, in our previous small-sample study, the rate of covered metallic ureteral stent migration was also correlated with the etiology of ureteral stricture. The stent migration rate of the covered metallic ureteral stent for ureteral stricture after ureteroscopic lithotripsy was 3.23% (1/31).^[6]^ In the study of the covered metallic ureteral stent for the treatment of ureteroileal anastomotic stricture, no stent migration occurred in all 8 patients at a mean follow-up of 9.8 months.^[21]^ Our covered metallic ureteral stent migration rate (25%) is similar to a recent study using a covered metallic ureteral stent to treat nonmalignant refractory ureteral stricture with a stent migration rate of 26.67% (4/15).^[5]^ However, our covered metallic ureteral stent migration rate is higher than a previous study using a covered metallic ureteral stent to treat recurrent ureteral stricture after ureteroplasty with a stent migration rate of 12.5% (3/24).^[25]^The previous study includes a total of 24 patients with recurrent stricture after ureteroplasty (pyeloplasty 14 patients, ureteroureterostomy 5 patients, ileal flap ureteroplasty 1 patient, buccal mucosa graft ureteroplasty 2 patients, and ureteral bladder replantation 2 patients). This suggests that the length, severity, and location of ureteral stricture may also affect the clinical outcome of the covered metallic ureteral stent. The higher rate of stent migration in our study may be due to the unique ureteral stricture characteristics of recurrent UPJO as compared to the recurrent ureteral stricture after other ureteroplasty. First, the ureteral stricture of recurrent UPJO patients is generally shorter and mild luminal stricture, therefore, the covered metallic ureteral stent which is fixed by self-expanding can easily migrate in this kind of stricture. In our study, 75% (15/20) of patients with recurrent UPJO had a ureteral stricture of ≤2 cm (Table 1). However, to our knowledge, there are no data that compares the effect of ureteral stricture length on the clinical outcome of covered metallic ureteral stents. Second, the ureteral stricture of recurrent UPJO is close to the wide renal pelvis. When the renal pelvis and ureter peristaltically move, the stent will be squeezed upward. Then, if the head of the stent protrudes too long into the renal pelvis, the covered metallic ureteral stent is more likely to migrate toward the renal pelvis. Therefore, we are trying to minimize the length of the stent head in the renal pelvis to reduce the stent migration rate.

Stent migration is a troublesome problem for both integral and segmental ureteral stents. Nowadays, J-shaped structures were introduced to the integral ureteral stents at the proximal and distal ends to prevent migration. However, the peristalsis of the ureter can still squeeze out the integral ureteral stent with a J-shaped structure, particularly when the ureteral stents are made from softer materials.^[24]^ For segmental ureteral stents, there is no effective way to completely prevent stent migration. Therefore, there are promising means to solve segmental ureteral stent migration by modifying the surface of the stent or improving the fixation of the stent in the future.

In addition to the stent migration, 1 patient was changed to nephrostomy because of stent encrustation, and another patient was changed to double-J ureteral stent because of stent infection. Although the covered metallic ureteral stent has a 35% (7/20) long-term stent-related complication rate and 15% (3/20) treatment failure possibility in the long-term maintenance treatment of recurrent UPJO (Tables 3 and 4), this treatment can still be used as the long-term maintenance treatment for recurrent UPJO and displayed a promising future. On the one hand, patients with complications in this study can be resolved by symptomatic treatment, stent adjustment, stent replacement, or stent removal. On the other hand, the success rate of long-term maintenance treatment of recurrent UPJO with covered metallic ureteral stent can still be further increased with the optimization of the material and design of the covered metallic ureteral stent and the clarification of the risk factors for failure of maintenance treatment of recurrent UPJO with a covered metallic ureteral stent.

Finally, this research has certain limitations. First, this is a retrospective study with a small sample size (20 cases), hence, the conclusions need to be confirmed by prospective randomized studies including a higher number of patients. Second, the median follow-up time of this study is 27.00 (18.00) months, and thus further extending the follow-up time to clarify the feasibility of this treatment is necessary.

## 5. Conclusions

The new segmental-covered metallic ureteral stent can effectively treat recurrent UPJO, which can relieve hydronephrosis and improve patients’ quality of life. In addition, it is a new long-term maintenance treatment option for properly selected recurrent UPJO patients. However, further prospective studies including a higher number of patients are required to confirm our results.

## Author contributions

**Conceptualization:** Xinwei Tang, Mingrui Wang, Haopu Hu, Chin-hui Lai, Qi Wang, Kexin Xu, Tao Xu, Hao Hu.

**Data curation:** Xinwei Tang, Mingrui Wang, Haopu Hu, Chin-hui Lai, Qi Wang.

**Formal analysis:** Xinwei Tang, Qi Wang.

**Investigation:** Xinwei Tang, Mingrui Wang, Haopu Hu.

**Methodology:** Xinwei Tang, Hao Hu.

**Project administration:** Xinwei Tang, Chin-hui Lai.

**Resources:** Xinwei Tang, Mingrui Wang, Chin-hui Lai, Qi Wang, Kexin Xu, Tao Xu, Hao Hu.

**Supervision:** Xinwei Tang, Hao Hu.

**Validation:** Xinwei Tang, Chin-hui Lai, Qi Wang, Kexin Xu, Tao Xu.

**Visualization:** Xinwei Tang.

**Writing – original draft:** Xinwei Tang, Mingrui Wang, Haopu Hu.

**Writing – review & editing:** Xinwei Tang, Mingrui Wang, Kexin Xu, Tao Xu, Hao Hu.

## Supplementary Material




