# Supplementary material for: Long-term maintenance treatment of recurrent ureteropelvic junction obstruction with covered metallic ureteral stent
Source: Medicine (Baltimore). 2023 Mar 31;102(13):e33363. doi: 10.1097/MD.0000000000033363 (PMC10063295; doi:10.1097/MD.0000000000033363)
Supplement: Supplementary file 1 [file medi-102-e33363-s001.pdf]

输尿管支架症状问卷

我们想了解放置支架管后您身体各个方面的健康状况以及支架管对您身体健康的影响。

请完成下面的问卷，该问卷包括几个部分。请回答各个部分中的所有问题。

（非常感谢您的帮助，请在 7 天内完成并寄回问卷）

请完成：

填写日期：□□／□□／□□

出生日期：□□／□□／□□

请寄回：

邮政编码：□□□□□□

住院号/门诊号：□□□□□□□/□□□□□□□□□□

您将看到某些问题询问您的某个症状是偶尔发生，有时发生或多数时候发生。

**偶尔发生**	=	**发生时间少于 1/3**
**有时发生**	=	**发生时间介于 1/3 到 2/3 之间**
**多数时候发生**	=	**发生时间超过 2/3**

尿路症状

请结合您在放置支架后出现的尿路症状回答问题。

请在每个结果的方框内打钩 ☑

请结合您在放置支架管后的体验

U1. 您在白天平均排尿间隔时间是？

不足 1 小时一次 □5
每小时一次 □4
每 2 小时一次 □3
每 3 小时一次 □2
每 4 小时或 4 小时以上一次 □1

U2. 您在夜间平均起床排尿多少次？

不排尿 □1
1 次 □2
2 次 □3
3 次 □4
4 次或 4 次以上 □5

U3. 您是否有过奔向厕所排尿？

从来没有 □1
偶尔出现（少于 1/3 次数）□2
有时出现（介于 1/3 次数到 2/3 次数之间）□3
多数时候出现（超过 2/3 次数）□4
总是出现 □5

U4. 是否在到达厕所前尿裤子？

从来没有 □1
偶尔出现 □2
有时出现 □3
多数时候出现 □4
总是出现 □5

U5. 是否在没有上厕所感觉时尿裤子？

从来没有 □1
偶尔出现 □2
有时出现 □3
多数时候出现 □4
总是出现 □5

U6. 多少次您在排尿后感觉到尿没有很好排净？

从来没有 □1
偶尔出现（少于 1/3 次数）□2
有时出现（介于 1/3 次数到 2/3 次数之间）□3
多数时候出现（超过 2/3 次数）□4
总是出现 □5

U7. 您是否在排尿时有烧灼感？

从来没有 □1
偶尔出现 □2
有时出现 □3
多数时候出现 □4
总是出现 □5

U8. 您多少次尿中带血？

从来没有 □1
偶尔出现 □2
有时出现 □3
多数时候出现 □4
总是出现 □5

U9. 您所见到尿中血色有多重？

见不到任何血色 □1
尿血色轻 □2
尿血色重 □3
尿血色重并且有血块 □4

U10. 总体上，您觉得尿路症状对您影响有多大？

没什么影响 □1
有一点 □2
中度 □3
比较大 □4
特别大 □5

U11. 如果您目前的与支架管有关的尿路症状伴随您下半生，您会觉得怎么样？

高兴 □1
满意 □2
大体满意 □3
感觉复杂（满意与不满意几乎各占一半） □4
大体不满意 □5
不满意 □6
糟糕透顶 □7

请前往下一部分

躯体疼痛（女性回答）

该部分了解您与支架管有关的**躯体疼痛或不适**。
请结合您在<u>放置支架管后</u>的体验。

P1. **您是否有与支架管相关的躯体疼痛或不适？**

有 □1，请前往问题 P2

没有 □2，请前往下一部分了解整体健康（忽略问题 P2 至 P9）

P2. 结合下面您躯体的示意图，请**在您出现与支架管相关的疼痛或不适（比如,在日常活动时，任何时间排尿时出现的疼痛或不适）的典型部位标记（×）或涂上阴影。**

如您的疼痛部位不止一个，请给**每个疼痛部位分别作出标记**。

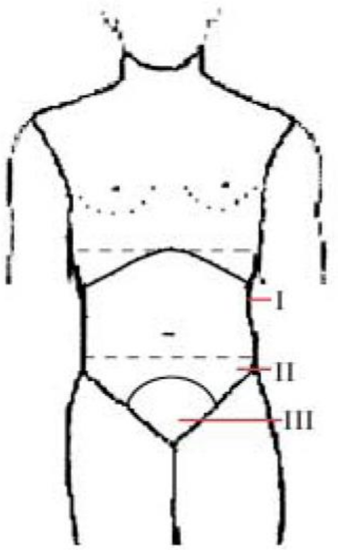

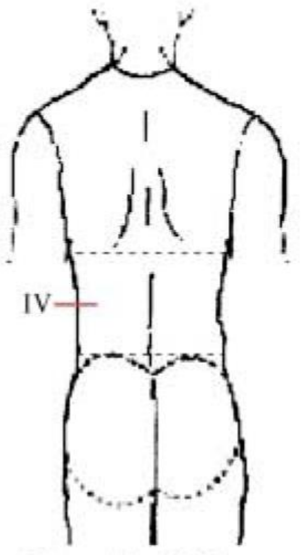

前面观 **后面观**

数字Ⅰ-Ⅳ代表以下区域左右两侧

Ⅰ-肾前面和侧面 Ⅲ-膀胱区
Ⅱ-腹股沟区 Ⅳ-肾后面（腰部）
其他需要标记的部位**请用○标记**并写下部位的名字

P3. 请在<u>下方反映您与支架管有关疼痛或不适程度的标尺的相应位置标记（×）</u>。如果疼痛或不适的部位不止一个且程度不同，**请为每个部位分别作出标记**，并写出上图所示各个部位的相应数字编号。

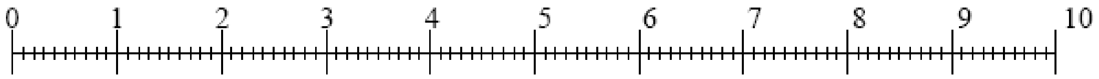

无疼痛或不适 **疼痛无法忍受**

躯体疼痛（男性回答）

该部分了解您**与支架管有关的躯体疼痛或不适**。
请结合您**放置支架管后**的体验。

P1. **您是否有与支架管相关的躯体疼痛或不适？**

有 □1，请前往问题 P2

没有 □2，请前往下一部分了解整体健康（忽略问题 P2 至 P9）

P2. 结合下面您躯体的示意图，**请在您出现与支架管相关的疼痛或不适（比如,在日常活动时，任何时间排尿时出现的疼痛或不适）的典型部位标记（×）或涂上阴影。**

如您的疼痛部位不止一个，请给每个疼痛部位分别作出标记。

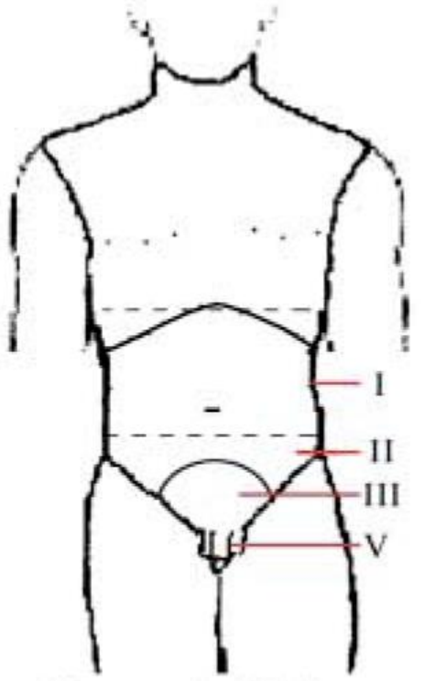

前面观

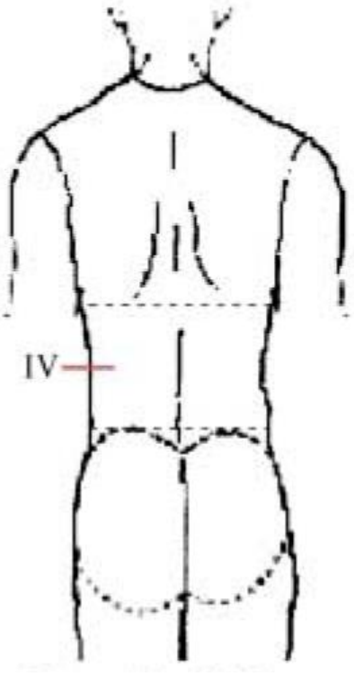

后面观

数字Ⅰ-Ⅴ代表以下区域左右两侧

Ⅰ-肾前面和侧面	Ⅳ-肾后面（腰部）
Ⅱ-腹股沟区	Ⅴ-阴茎
Ⅲ-膀胱区	其他需要标记的部位**请用○标记**并写下部位的名字

P3. 请在**下方反映您与支架管有关疼痛或不适程度的标尺的相应位置标记**（×）。如果疼痛或不适的部位不止一个且程度不同，请为**每个部位分别作出标记**，并写出上图所示各个部位的相应数字编号。

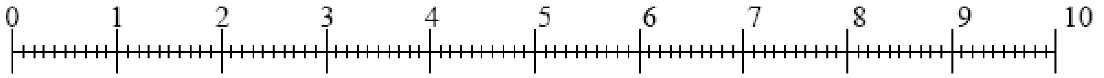

无疼痛或不适 **疼痛无法忍受**

P4. 下列哪一条最能反映您体力活动时出现的与支架管相关的疼痛或不适？

我在体力活动时**没有感到任何疼痛**或不适 □1

我仅在**剧烈活动**（如剧烈运动，提举重物）时出现疼痛或不适 □2

我在**中等程度活动**（如步行几百米，开车）时，但不是基本活动时，出现疼痛或不适 □3

我甚至在日常生活的**基本活动**（如室内走动，穿衣）时出现疼痛 □4

我即使**休息时**也出现疼痛 □5

P5. 与支架管相关的疼痛或不适是否影响您的睡眠？

从未影响 □1

偶尔 □2

有时 □3

多数时候 □4

总是 □5

P6. 您排尿时是否出现与支架管相关的疼痛或不适？

从未出现 □1

偶尔 □2

有时 □3

多数时候 □4

总是 □5

P7. 您排尿时是否出现肾脏区域的疼痛或不适？

没有 □1

有 □2

P8. 您多少次需要用止痛药控制与支架管相关的疼痛或不适？

从不需要 □1

偶尔 □2

有时 □3

多数时候 □4

总是 □5

P9. 总体上，与支架管相关的疼痛或不适（不同于其他症状）对您生活的影响程度有多大？

没什么影响 □1

有一点 □2

中度 □3

比较大 □4

特别大 □5

请前往下一部分

总体健康：

放置支架管后：

G1. 您在进行轻度体力活动（如短途步行，开车）时是否有困难？

通常没有困难 □1
通常有些困难 □2
通常有很大困难 □3
通常因为支架管而不去做 □4
总是有困难 □5

G2. 您在进行重体力活动（如剧烈运动，提举重物）时是否有困难？

通常没有困难 □1
通常有些困难 □2
通常有很大困难 □3
通常因为支架管而不去做 □4
总是有困难 □5

G3. 您是否感到疲倦或疲乏？

从未感到 □1
偶尔（少于 1/3 次数）□2
有时（介于 1/3 次数到 2/3 次数之间）□3
多数时候（超过 2/3 次数）□4
总是 □5

G4. 您是否感到平静和安宁？

总是 □1
多数时候（超过 2/3 次数）□2
有时（介于 1/3 次数到 2/3 次数之间）□3
偶尔（少于 1/3 次数）□4
从未感到 □5

G5. 您是否能享受您的社交生活（外出，朋友聚会，诸如此类）？

总是 □1
多数时候 □2
有时 □3
偶尔 □4
从未享受到 □5

G6. 您是否需要家人或朋友的额外帮助？

从不需要 □1
偶尔 □2
有时 □3
多数时候 □4
总是 □5

请前往下一部分

工作表现

W1. 考虑到您的工作状况，您是

全职工作 □1
兼职工作 □2
因健康原因退休 □3
因其他原因未工作（请具体说明）□7 ______________________________
学生 □4
失业，正在找工作 □5
因其他原因退休（包括年龄）□6

W2. 放置支架管后，有多少天您因支架管相关的症状需要终日或一天中大多数时间卧床？

□□天

W3. 放置支架管后，有多少半天以上您因支架管相关的症状而减少常规的活动？

□□半天

如您有稳定工作请回答下列问题（W4-W7）

（否则忽略问题 W4-W7）

W4. a）职称或描述您的角色：__________

b）您是 雇员 □1 雇主 □2 个体户 □3

<u>如您在放置支架管后继续从事工作请回答下列问题，</u>

W5. 您是否因支架管相关的症状而缩短工作时间或增加休息次数？

从不 □1
偶尔 □2
有时 □3
多数时候 □4
总是 □5

W6. 您是否继续从事平常的工作，但因支架管相关的症状作了某些改变？

从未改变 □1
偶尔 □2
有时 □3
多数时候 □4
总是 □5

W7. 您工作的小时数是否和从前相同？

总是不同 □5
偶尔 □4
有时 □3
多数时候 □2
总是相同 □1

请前往下一部分

性生活

请结合您**放置支架管后**的经历在相应的答案方框内打钩

S1. 目前，您是否有活跃的性生活？

没有 □1，请回答问题 S2 并前往下一部分（忽略问题 S3 和 S4）。

有 □2，请前往问题 S3（忽略问题 S2）。

S2. (i) 如没有性生活，停止性生活有多久？

放置支架管后 □1

放置支架管前 □0

(ii) 并且，为什么停止性生活？

因为支架管相关的问题 □10

不尝试任何性活动 □0

某些其他原因-与支架管相关症状无关 □0

（忽略问题 S3-S4）

仅当您在问题 S1 选择“有”时才需要请您回答问题 S3 和 S4。

请结合您放置支架管后的经历。

S3. 您在性交时是否感到疼痛？

一点没有 □1

轻度 □2

中度 □3

重度 □4

特别严重 □5

S4. 您对您性生活的满意程度如何？

非常满意 □1

满意 □2

不好说 □3

不满意 □4

很不满意 □5

请前往下一部分

其他问题：

以下问题了解您在放置支架管后的体验。请在反映您体验的合适方框内打钩。

A1. 有多少次您觉得您可能患上尿路感染（如发热，感觉排尿不适及疼痛）？

从未感到 □1
偶尔 □2
有时 □3
多数时候 □4
总是 □5

A2. 您是否需要因放置支架管而服用抗生素（放置支架管时给予的那个疗程抗生素请不要考虑在内）？

不需要 □1
一个疗程 □2
两个疗程 □3
三个以上疗程 □4

A3. 您是否因为任何支架管相关的问题而需要寻求医务人员（如社区医生，护士）的帮助？

从未需要 □1
一次 □2
两次 □3
三次以上 □4

A4. 您是否因支架管相关的问题而需要去医院？

从未需要 □1
一次 □2
两次 □3
三次以上 □4

一般问题. 将来，如您需要再次放置支架管，您会觉得如何？

高兴 □1
满意 □2
大体满意 □3
感觉复杂（满意与不满意几乎各占一半）□4
大体不满意 □5
不满意 □6
糟糕透顶 □7

其他问题. 如您想对本问卷或对您的任何症状作出评论，请使用下方空白处？

非常感谢您的帮助
所有信息都将保密
